# Splenic Infarction at the Crossroads of Hematologic and Cardioembolic Risk

**DOI:** 10.7759/cureus.88032

**Published:** 2025-07-15

**Authors:** George K Annan, Enoch Enninful, Nana Dwommoh, Chinenye Egwuonwu, Sudeep Chapagain

**Affiliations:** 1 Internal Medicine, Piedmont Athens Regional Medical Center, Athens, USA; 2 Plastic and Reconstructive Surgery, Korle Bu Teaching Hospital, Accra, GHA; 3 Anesthesia and Critical Care, Komfo Anokye Teaching Hospital (KATH), Kumasi, GHA

**Keywords:** atrial fibrillation, chronic myeloid leukemia, cml, hyperleukocytosis, splenic infarction, thromboembolic complications

## Abstract

Splenic infarction is an uncommon but clinically relevant complication of both hematologic malignancies and cardioembolic disorders. Chronic myeloid leukemia (CML), particularly when associated with hyperleukocytosis and thrombocytosis, contributes to a prothrombotic state. Atrial fibrillation, even in its paroxysmal form, is a well-established risk factor for systemic embolism. When these conditions coexist, thromboembolic risk is significantly elevated, creating complex management challenges due to concurrent bleeding risks.

We present a 76-year-old woman with T315I BCR-ABL1 chronic-phase CML, paroxysmal atrial fibrillation, and hypertension who presented with acute left upper quadrant abdominal pain. Imaging studies confirmed splenic infarction. Laboratory findings revealed marked leukocytosis and thrombocytosis. She was treated with intravenous fluids, analgesia, and anticoagulation; initially with heparin infusion, later transitioned to apixaban. Concurrently, disease-directed therapy with asciminib and hydroxyurea led to a partial hematologic response. At three-month follow-up, she remained free of recurrent thrombosis, bleeding, or abdominal symptoms.

This case highlights the multifactorial etiology of splenic infarction in patients with overlapping hematologic and cardiovascular risk factors. It underscores the importance of timely imaging for diagnosis and the delicate balance of anticoagulation in the setting of malignancy-associated thrombocytosis. Individualized, multidisciplinary management and careful long-term follow-up are essential to optimize outcomes in this complex patient population.

## Introduction

Splenic infarction results from interruption of the splenic arterial blood flow, most commonly from occlusion of the splenic artery or its branches. Splenic infarction is rare overall, with an estimated incidence of 0.016% of hospital admissions in a 10-year retrospective study at an academic general hospital [1]. While uncommon, it is a recognized complication of thromboembolic disease and hematologic malignancies, with other etiologies being infectious and infiltrative diseases [1,2].

The cardiovascular causes of splenic infarction are primarily cardioembolic, with atrial fibrillation consistently identified as the leading risk factor, especially in older adults, due to its high propensity for left atrial thrombus formation and systemic embolization [1-3]. Other cardiovascular risk factors for splenic infarction are valvular heart disease and infective endocarditis [1-3].

Chronic myeloid leukemia (CML) is a clonal myeloproliferative neoplasm characterized by the presence of the BCR-ABL1 fusion gene, which results from a reciprocal translocation between chromosomes 9 and 22, creating the Philadelphia chromosome. This fusion gene encodes a constitutively active tyrosine kinase that drives leukemic proliferation. CML can present in the chronic, accelerated, or blast phases [4,5]. Patients in the chronic phase may remain stable on tyrosine kinase inhibitors (TKIs), but those with high-risk features such as significant basophilia or blast presence are at greater risk of disease progression and complications, including thrombotic events due to greater disease burden and cellular abnormalities [5-7]. CML contributes to thrombosis via increased and activated blood cells, chronic inflammation, endothelial activation, and direct procoagulant activity of leukemic cells and their progeny, all of which are implicated in the pathogenesis of complications such as splenic infarction [8-10].

The coexistence of CML and atrial fibrillation may significantly elevate thromboembolic risk through overlapping yet distinct mechanisms. The true magnitude of this compounded risk remains underexplored in clinical research [11-13]. To our knowledge, splenic infarction in the setting of coexisting paroxysmal atrial fibrillation and high-risk chronic-phase CML has been rarely documented. This case adds to the limited literature on compounded thrombotic risk in patients with overlapping hematologic and cardiovascular disorders.

Additionally, current stroke and bleeding risk stratification models [e.g., Congestive Heart Failure, Hypertension, Age ≥75 years, Diabetes Mellitus, Stroke/Transient Ischemic Attack/Thromboembolism, Vascular Disease, Age 65-74 years, and Sex Category (Female) (CHA₂DS₂-VASc), Hypertension, Abnormal Liver/Renal Function, Stroke, Bleeding History or Predisposition, Labile International Normalized Ratio, Elderly (Age >65), Drugs/Alcohol Concomitantly (HAS-BLED)] may not be fully reliable in cancer populations, including those with CML, as they fail to account for malignancy-specific risks such as cytopenias, chemotherapy effects, and drug interactions. This complicates individualized anticoagulation decisions and underscores the need for cancer-specific risk assessment models [11-16].

Patients with CML face a heightened risk of bleeding when on anticoagulation, driven by disease-related factors like thrombocytopenia and platelet dysfunction, as well as effects of cancer therapies such as tyrosine kinase inhibitors. Standard tools like HAS-BLED may underestimate this risk. Data on anticoagulation safety in CML, particularly with direct oral anticoagulants, remain limited, highlighting the need for further research and individualized risk assessment [17-21].

We report a case of splenic infarction in a patient with high-risk chronic-phase CML and paroxysmal atrial fibrillation, highlighting the compounding risk of thrombosis and the importance of prompt, multidisciplinary management.

## Case presentation

A 76-year-old woman with a history of T315I BCR-ABL1 chronic-phase CML, hypertension, and paroxysmal atrial fibrillation not on prophylactic anticoagulation, presented with a day’s history of acute-onset, severe, sharp, and non-radiating left upper quadrant abdominal pain. She denied trauma, blurred vision, headache, confusion, fever, chills, urinary or gastrointestinal symptoms.

Notably, she had been discharged five days earlier following hospitalization for a urinary tract infection, during which significant hyperleukocytosis (white cell count 108 ×10⁹/L) and thrombocytosis (platelets 1135 ×10⁹/L) prompted concern for CML progression as she had defaulted on her asciminib for about six months. Bone marrow biopsy revealed 32% basophils (≥20%) consistent with high-risk CML [22]. There were 5% blasts. She was restarted on asciminib. Hydroxyurea and allopurinol were added for cytoreduction and tumor lysis syndrome prophylaxis, respectively. At that time, prophylactic anticoagulation was not initiated due to the risk of bleeding and lack of recent atrial fibrillation episodes. She was discharged with a planned outpatient follow-up within a week.

**Figure 1:**
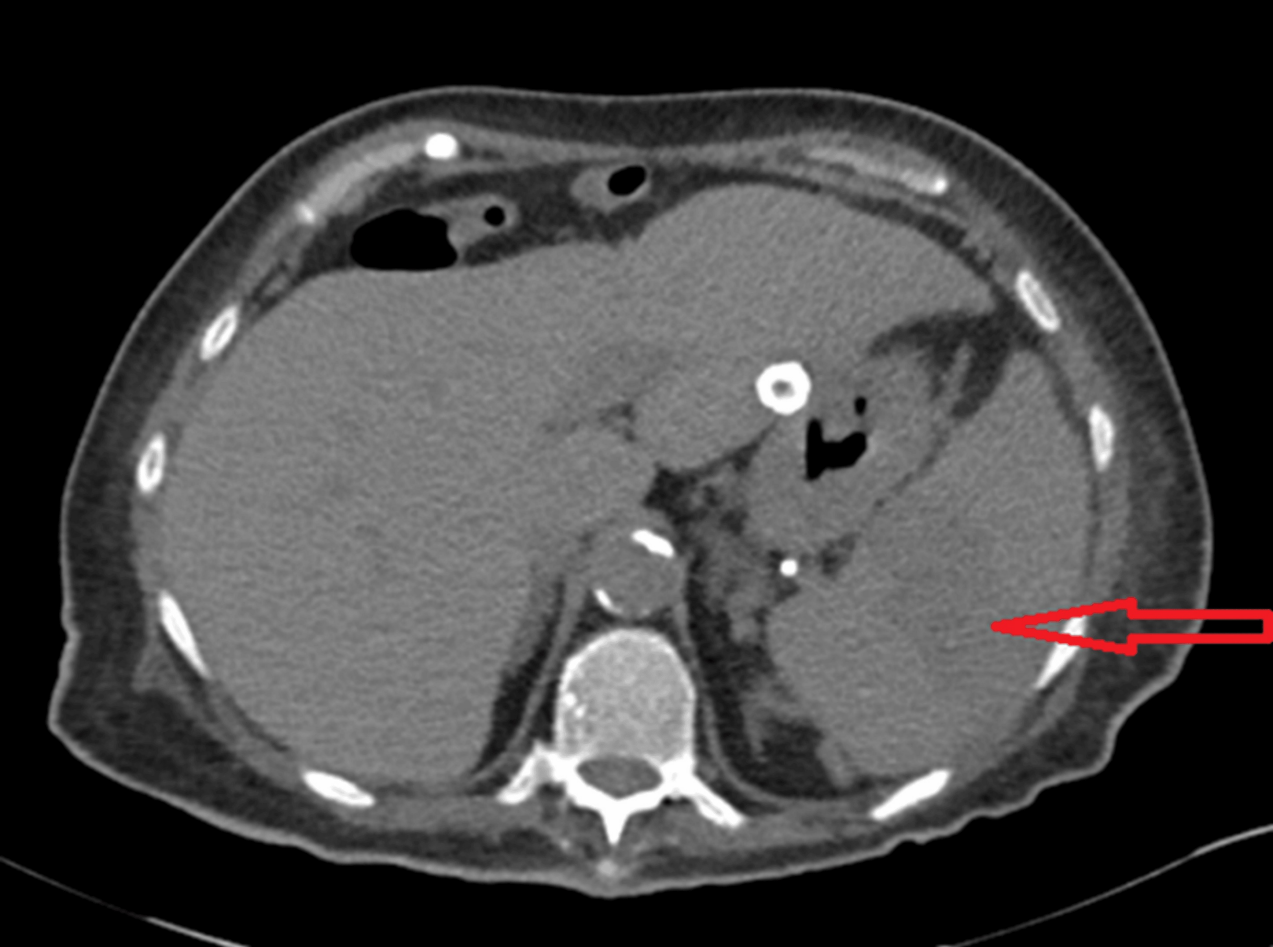
CT abdomen showing splenic infarction in a patient with chronic myeloid leukemia (CML) Axial Computed Tomography image of the abdomen demonstrating a linear area of hypoattenuation in the central spleen (arrow), consistent with splenic infarction. This radiologic finding confirmed the suspected diagnosis and prompted initiation of systemic anticoagulation after risk-benefit assessment. The spleen appears enlarged, a common finding in chronic myeloid leukemia due to extramedullary hematopoiesis and leukemic infiltration.

**Figure 2:**
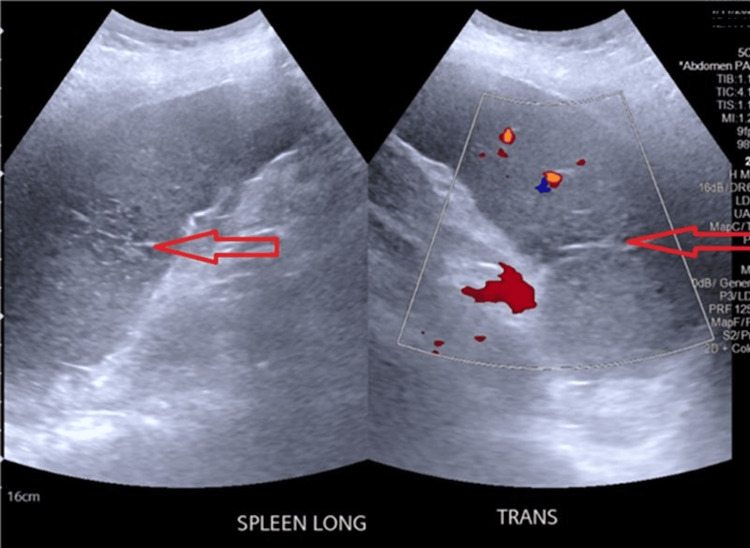
Ultrasound of the spleen demonstrating features of splenic infarction Ultrasound image of the spleen showing a focal, wedge-shaped hypoechoic region centrally, without detectable color or spectral Doppler flow, findings consistent with splenic infarction. The splenic hilar vessels are patent, and there is no perisplenic fluid collection. The ultrasound was used to confirm the CT findings and to exclude other structural abnormalities or abscess formation. The spleen is enlarged, measuring 15.7 cm in length.

On presentation, her vital signs were stable with a regular heart rate. Physical examination revealed tenderness in the left upper quadrant. Laboratory evaluation showed white cell count 74.4 ×10⁹/L, hemoglobin 11.3 g/dL, and platelet count 1183 ×10⁹/L. Laboratory findings are summarized in Table 1 below. CT abdomen (Figure 1) demonstrated linear hypoattenuation within the spleen, and splenic ultrasound (Figure 2) confirmed splenic infarction. Other than mild mitral valve stenosis, the echocardiogram did not show evidence of vegetations, and blood culture was unremarkable.

**Table 1:** Laboratory findings on prior admission, presentation, and discharge Selected hematologic parameters showing trends over time in a 76-year-old female with chronic-phase chronic myeloid leukemia (CML) and splenic infarction. The table highlights persistent leukocytosis and thrombocytosis during the initial phases of presentation with improvement following cytoreductive therapy. Reference ranges are provided for comparison.

Parameter	Value on previous admission	Value at Presentation	Value at Discharge	Reference Range
White blood cell count	108 × 10⁹/L	74.4 × 10⁹/L	11 × 10⁹/L	4.0 – 11.0 × 10⁹/L
Hemoglobin	11.5 g/dL	11.3 g/dL	10.9 g/dL	12.0 – 16.0 g/dL
Platelets	1135 × 10⁹/L	1183 × 10⁹/L	776 × 10⁹/L	150 – 400 × 10⁹/L
Basophils (bone marrow)	32%			<1%
Blasts (bone marrow)	5%			<5%

The patient was admitted for intravenous fluids, analgesia, and anticoagulation with a heparin infusion. She continued treatment with asciminib and hydroxyurea for her chronic-phase CML with high-risk features. Telemetry monitoring documented four episodes of paroxysmal atrial fibrillation over two days, each lasting less than 10 minutes. Given her high risk of future thromboembolism (CHA₂DS₂-VASc score of 6: age, sex, hypertension, thromboembolism), and moderate risk of bleeding (HAS-BLED score of 2: predisposition to bleeding, age), she was discharged on prophylactic apixaban 5mg twice daily after an extensive risk versus benefit discussion. A close follow-up was arranged. At the time of discharge, her white cell count and platelets were 11×10⁹/L and 776×10⁹/L respectively, indicating partial hematologic response. On re-evaluation at three months, the patient exhibited no signs of recurrent thrombosis, bleeding, or residual abdominal discomfort. Imaging was not repeated as there was no clinical indication.

## Discussion

Splenic infarction is a rare clinical entity resulting from interruption of splenic arterial perfusion, most commonly due to thromboembolic occlusion [1,2]. The clinical presentation may mimic other causes of acute abdominal pain and is often underdiagnosed without imaging. In this case, the patient presented with acute left upper quadrant pain, ultimately attributed to splenic infarction secondary to overlapping thrombotic risks from chronic-phase CML with high-risk features and paroxysmal atrial fibrillation. Blood cultures were negative, there were no signs of systemic infection, and CT imaging did not reveal aneurysmal changes or signs of vasculitis. These alternative causes were therefore considered unlikely.

CML contributes to a prothrombotic state through several mechanisms, including increased blood viscosity from hyperleukocytosis and thrombocytosis, abnormal platelet function, and direct procoagulant activity of leukemic cells and their microparticles [8-10]. These effects are amplified in patients with high-risk disease features such as bone marrow basophils ≥20%, which was present in our patient [5-7]. Such hematologic derangements can cause vascular congestion and microvascular occlusion, particularly in organs like the spleen, which is already susceptible due to its unique low-pressure circulation.

Simultaneously, paroxysmal atrial fibrillation adds an embolic dimension to the patient’s thrombotic risk. Paroxysmal atrial fibrillation increases the risk of systemic thromboembolism, although the risk is less when compared to sustained atrial fibrillation [23,24]. In the context of high leukocyte and platelet counts, even transient episodes of atrial fibrillation may substantially increase thromboembolic potential. Her mild mitral stenosis was considered hemodynamically insignificant and not a major contributor to embolic risk, particularly as there were no vegetations and blood cultures were negative.

This case demonstrates the heightened risk that arises when two potent thrombotic mechanisms, hematologic malignancy and atrial fibrillation, coexist. Management involved supportive care with intravenous fluids and analgesia, along with systemic anticoagulation, initially with heparin and later transitioned to apixaban. The decision to initiate anticoagulation was guided not only by the confirmed infarct but also by the patient’s multiple thromboembolic risk factors.

The increased bleeding risk associated with anticoagulation was appropriately acknowledged and discussed with the patient. No studies have specifically evaluated the impact of splenic infarct size on CML outcomes, and current evidence focuses on overall spleen size rather than infarct characteristics [25]. Close follow-up was arranged to monitor for bleeding and further thrombotic events. This case highlights the need for a personalized, multidisciplinary approach in patients with overlapping prothrombotic conditions.

## Conclusions

Splenic infarction is a rare but clinically significant complication that may arise in patients with chronic myeloid leukemia, especially those with high-risk hematologic features. The presence of additional embolic risk factors, such as paroxysmal atrial fibrillation, further amplifies the potential for thrombotic events. Clinicians should maintain a high index of suspicion in patients with hematologic malignancies presenting with abdominal pain, given the potential for diagnostic delay due to nonspecific symptoms. In this case, prompt imaging was crucial in confirming the diagnosis and guiding the initiation of systemic anticoagulation.

Management requires individualized anticoagulation strategies based on factors such as platelet count, atrial fibrillation burden, bleeding risk, and overall disease status. Long-term follow-up is essential to monitor for recurrent thrombotic events, bleeding complications, and disease progression. The patient demonstrated clinical and hematologic improvement with cytoreductive therapy and anticoagulation, with no recurrent events at three-month follow-up.

Further research is needed to better quantify these overlapping risks and to develop cancer-specific risk assessment tools that guide anticoagulation in this unique patient population. Prospective studies and registry data could help inform future management algorithms and improve clinical outcomes.
